# Supplementary material for: Asynchronous magnetic resonance elastography: Shear wave speed reconstruction using noise correlation of incoherent waves
Source: Magn Reson Med. 2022 Oct 27;89(3):990–1001. doi: 10.1002/mrm.29502 (PMC9792433; doi:10.1002/mrm.29502)
Supplement: Supplementary file 1 — DATA S1 MATLAB code used to generate the numerical simulations of Figure 1 [file MRM-89-990-s002.zip › k-Wave/helpfiles/example_tvsp_homogeneous_medium_monopole.html]

Monopole Point Source In A Homogeneous Propagation Medium Example (k-Wave)


# Monopole Point Source In A Homogeneous Propagation Medium Example

This example provides a simple demonstration of using k-Wave for the simulation and detection of a time varying pressure source within a two-dimensional homogeneous propagation medium. It builds on the Homogeneous Propagation Medium and Recording The Particle Velocity examples.

- Open the file in the MATLAB Editor
- Run the file in MATLAB

## Contents

- Defining the time varying pressure source
- Source filtering
- Running the simulation

## Defining the time varying pressure source

In the Initial Value Problem examples, the initial pressure distribution is defined by assigning the source values to `source.p0`. To define a time varying pressure source, both a source mask (which defines which grid points belong to the source) and a time varying source input must be assigned. The source mask is defined by assigning a binary matrix (i.e., a matrix of 1's and 0's with the same dimensions as the computational grid) to `source.p_mask`, where the 1's represent the grid points that form part of the source. The time varying input signal is then assigned to `source.p` which is indexed as `source.p(source_point_index, time_index)`. The source can have any number of time points (it doesn't need to match `kgrid.Nt`) and can be defined as either a single time series (in which case the same time series is applied to all of the source points), or a matrix of time series following the source points using MATLAB's column-wise linear matrix index ordering. For example, if `source.p_mask` is defined as

```
source.p_mask =
     0     1     0
     1     0     1
     1     0     1
     0     1     0
```

the ordering of the source points within `source.p(source_point_index, time_index)` would follow

```
     0     3     0
     1     0     5
     2     0     6
     0     4     0
```

In this example a sinusoidal input is assigned to a single source element to create an acoustic monopole. The remaining input structures are defined in the same way as in previous examples.

```
% define a single source point
source.p_mask = zeros(Nx, Ny);
source.p_mask(end - Nx/4, Ny/2) = 1;

% define a time varying sinusoidal source
source_freq = 0.25e6;   % [Hz]
source_mag = 2;         % [Pa]
source.p = source_mag * sin(2 * pi * source_freq * kgrid.t_array);
```

## Source filtering

The temporal sampling frequency of the input and output signals is dictated by the size of the time step, `kgrid.dt`. This means the highest frequency that can be represented in a time varying pressure or velocity input is the Nyquist limit of `1/(2*kgrid.dt)`. However, the highest temporal frequency that can be represented on the spatial grid is given by the Nyquist limit of `medium.sound_speed/(2*dx)` or `CFL/(2*kgrid.dt)`. For most simulations, the CFL number will be less than 1 (the `makeTime` method of the `kWaveGrid` class uses a CFL of 0.3 by default). This means it is possible to define time varying pressure or velocity input signals that contain frequencies that cannot be represented on the grid, so care must be taken that maximum frequency supported by the grid is not exceeded.

The maximum frequency supported by the grid is reported on the command line at the beginning of each simulation. Input signals can be automatically restricted to the range of supported frequencies by using the function `filterTimeSeries`. This applies a finite impulse response (FIR) filter designed using the Kaiser windowing method. The filter can be set to either zero or linear phase as required.

```
% filter the source to remove high frequencies not supported by the grid
source.p = filterTimeSeries(kgrid, medium, source.p);
```

## Running the simulation

In the Recording The Particle Velocity Example, an input for `sensor.record` was defined to record both the pressure and velocity fields at the sensor points defined by `sensor.mask`. In this example, the final pressure field within the computational domain is also returned by setting `sensor.record` to `{'p', 'p_final'}`. Unlike the outputs for pressure and velocity, the outputs for `'p_final'` and `'u_final'` are returned over the complete computational grid. In 2D they are indexed as `(nx, ny)`. A plot of the input time series driving the source point along with the acoustic pressure recorded at the sensor point and the final pressure field is given below.

```
% define the acoustic parameters to record
sensor.record = {'p', 'p_final'};

% run the simulation
sensor_data = kspaceFirstOrder2D(kgrid, medium, source, sensor);
```
